# Exploring the potential relationship between frozen shoulder and Dupuytren’s disease through bioinformatics analysis and machine learning

**DOI:** 10.3389/fimmu.2023.1230027

**Published:** 2023-08-31

**Authors:** Yulong Ouyang, Shuilin Chen, Yuanqing Tu, Ting Wan, Hao Fan, Guicai Sun

**Affiliations:** ^1^ Department of Orthopedics, The First Affiliated Hospital of Nanchang University, Nanchang, Jiangxi, China; ^2^ The First Clinical Medical College, Nanchang University, Nanchang, Jiangxi, China

**Keywords:** frozen shoulder, Dupuytren’s disease, diagnosis, machine learning, immunocyte infiltration

## Abstract

**Background:**

Frozen shoulder (FS) and Dupuytren’s disease (DD) are two closely related diseases, but the mechanism of their interaction is unknown. Our study sought to elucidate the molecular mechanism of these two diseases through shared gene and protein interactions.

**Methods:**

GSE75152 and GSE140731 data were downloaded from the Gene Expression Omnibus (GEO) database, and shared genes between FS and DD were selected by using R packages. Then, we used Cytoscape software and the STRING database to produce a protein−protein interaction (PPI) network. Important interaction networks and hub genes were selected through MCODE and cytoHubba algorithms. To explore the potential mechanisms of the development of the two diseases, the hub genes were further enriched by GO and KEGG analyses. We predicted the transcription factors (TFs) of hub genes with Transcriptional Regulatory Relationships Unraveled by Sentence-based Text mining (TRRUST). Moreover, we identified candidate genes for FS with DD with cytoHubba and machine learning algorithms. Finally, we analyzed the role of immunocyte infiltration in FS and constructed the relationship between candidate genes and immunocytes in FS.

**Results:**

We identified a total of 321 shared genes. The results of GO and KEGG enrichment of shared genes showed that extracellular matrix and collagen fibril tissue play a certain role in the occurrence and development of disease. According to the importance of genes, we constructed the key PPI network of shared genes and the top 15 hub genes for FS with DD. Then, we predicted that five TFs are related to the hub genes and are highly expressed in the FS group. Machine learning results show that the candidate genes POSTN and COL11A1 may be key for FS with DD. Finally, immune cell infiltration revealed the disorder of immunocytes in FS patients, and expression of candidate genes can affect immunocyte infiltration.

**Conclusion:**

We identified a PPI network, 15 hub genes, and two immune-related candidate genes (POSTN and COL11A1) using bioinformatics analysis and machine learning algorithms. These genes have the potential to serve as diagnostic genes for FS in DD patients. Furthermore, our study reveals disorder of immunocytes in FS.

## Introduction

Frozen shoulder (FS) is a common joint disease that causes pain and stiffness (1). FS is the third most common cause of musculoskeletal disability in the United States and affects patients between the ages of 40 and 60 years, with a prevalence rate of 2% to 5% (2, 3). FS is typically classified as either primary or secondary FS (4). Primary FS usually occurs without a clear cause, whereas secondary FS is caused by trauma or immobilization (4). Although the disease is self-limited and shows some improvement after conservative treatment or surgical treatment, most patients who are followed up long term develop permanent disability (5, 6). However, the pathogenesis of FS is still unclear.

Studies have shown that FS may be associated with hyperthyroidism, hypothyroidism, diabetes, and Dupuytren’s disease (DD) (7). Among these diseases, DD and FS have very similar histopathological and immunocytochemical characteristics (8, 9). DD is one of the most widespread inherited connective tissue disorders and appears to be common in northern Europeans (10). The prevalence of DD ranges from 3% to 42% worldwide (11). DD is a common benign fibroproliferative condition (12). A fibrotic nodule over the palmar fascia is the most common symptom of the disorder. These bands have the ability to contract, leading to flexion contracture of the facet joints of the hand, known as Dupuytren’s contracture (DC) (13). However, the exact pathophysiology of DD is not known.

The unknown causes of FS and DD make treating patients difficult, and their high prevalence brings a great economic burden to society (14). Therefore, studying the pathogenesis is helpful and necessary for the development of disease treatments. Increasing evidence shows not only that there are some similar pathological features between DD and FS but also that there is a strong clinical correlation between them (12, 15). There may be a breakthrough in understanding the pathogenesis of these two diseases due to their similarity. However, little is known about the common characteristics of FS and DD based on gene regulatory mechanisms.

Recently, as sequencing technology and bioinformatics have developed, it has become possible to determine how diseases interact genetically (16, 17). Screening out hub genes and analyzing the correlation between them is very important for studying diseases. In this study, we aimed to uncover shared genes and clusters of coexpression between FS and DD, providing a relevant foundation for future research on the two diseases. We constructed a protein–protein interaction (PPI) network for the shared genes and identified hub and candidate genes by using cytoHubba and machine learning algorithms. We explored the relationship between transcription factors (TFs) and these diseases on the basis of hub genes. Moreover, we analyzed the role of immunocytes in FS patients and constructed the relationship between candidate genes and immunocytes in FS.

## Methods

### Dataset download and processing

We used the term “frozen shoulder” or “Dupuytren’s disease” to search for gene expression profiles in the Gene Expression Omnibus (GEO) (http://www.ncbi.nlm.nih.gov/geo/) database. GSE75152 and GSE140731 were obtained. For GSE75152, total mRNA was extracted from connective tissue in the hand and detected with the Illumina HumanHT-12 V3.0 expression beadchip (18). For GSE140731, total mRNA was extracted from tissue from the anterior capsule and rotator interval and detected using RNA sequencing (19). These data are all from humans. After downloading the two datasets, we preprocessed the data. Then, we performed log2 transformation for gene expression profiling and matched the probes to their gene symbols using the annotation document for the appropriate platforms. Finally, a gene matrix was obtained for subsequent analyses.

### Identification of shared genes

We identified differentially expressed genes (DEGs) in FS and DD (|log FC| > 0.585, adjusted *p*-value < 0.05). There was overlap between genes in the FS and DD modules with positive correlation coefficients, as assessed by the “VennDiagram” package (20). Functional enrichment was performed to analyze common DEGs and total DEGs in DD and FS via R packages (enrichplot, RColorBrewer, ComplexHeatmap, etc.).

### Physical protein interaction network

DD and FS shared genes were used to draw a physical interaction network with the STRING database (version 11.5). The interaction network was extracted, and the extracted network data were visualized by using Cytoscape software (version 3.9.1). To analyze the extracted network data using Cytoscape software, the MCODE algorithm and the cytoHubba algorithm were employed in the analysis of the PPI network and cluster analysis. Gene clusters with the top 15 hub genes were obtained. The interacting genes and functions of these genes were predicted, and related PPIs were generated in the GeneMANIA database (https://genemania.org/). Further enrichment of hub clusters was achieved through analysis of GO functional terms and KEGG pathway enrichment analysis.

### Construction of the transcriptional regulatory network

To identify substantial changes at the transcriptional level and to gain an in-depth understanding of the regulatory role of DEGs, we used the hub gene to introduce Transcriptional Regulatory Relationships Unraveled by Sentence-based Text mining (TRRUST, https://www.grnpedia.org/trrust/). Next, enrichment of hub genes was assessed to obtain corresponding TFs. Then, Cytoscape software was used to produce a TF regulatory network. Finally, differential expression of TFs in FS patients was analyzed to identify TFs that play a critical role in the disease.

### Machine learning

We used least absolute shrinkage and selection operator (LASSO) regression and support vector machine (SVM) learning to identify potential candidate genes related to the diagnosis of FS and DD. LASSO regression is a machine learning technique that combines variable selection and regularization, which can improve prediction accuracy (21). SVM learning is very powerful at recognizing subtle patterns in complex datasets (22). It aims to create a decision boundary between two classes such that labels from one or more feature vectors can be predicted (23). We used the “glmnet”, “e1071”, “kernlab”, and “caret” software packages of R software to carry out LASSO regression and SVM learning. The intersection of these two results can be used as candidate genes for diagnosis. To determine the importance of candidate genes in diagnosis, we used the “rms” R package to construct a nomogram. We further evaluated the prognostic value of the candidate genes by ROC analysis. The area under the ROC curve (AUC) and 95% confidence interval (CI) were obtained. An AUC value > 0.7 is considered to indicate good diagnostic efficacy.

### Immune infiltration analysis

We used the CIBERSORT tool to evaluate immunocyte infiltration based on gene expression profiles. A bar plot was used to visualize the proportion of immunocytes, while a violin plot was used to compare the proportions of these cells between the FS and control groups. We established the relationship between candidate genes and immunocytes by Spearman analysis and used a Lollipop plot to visualize their correlation.

### Statistical analysis

Data were analyzed with R 4.3.0 (https://www.rproject.org/), Cytoscape software (version 3.9.1), Perl 5.32.1 (https://www.perl.org), and R Bioconductor packages. All statistical *p*-values were two-sided, with *p* < 0.05 considered statistically significant.

## Results

### Identification of DEGs in DD and FS

**Figure 1:**
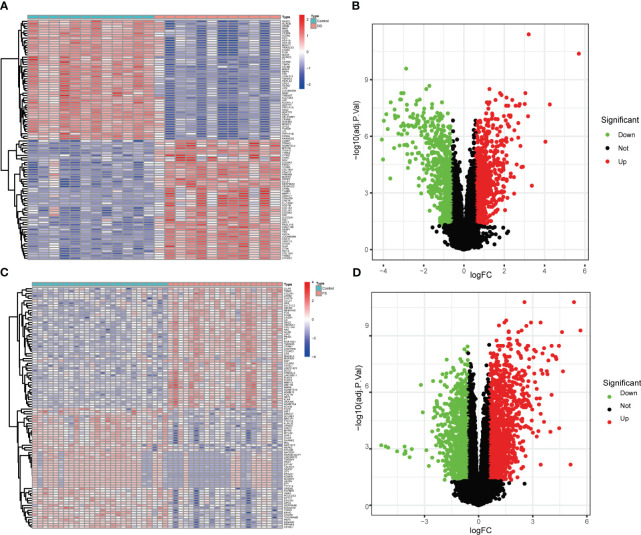
Volcano plot and heatmap show genetic differences between the two groups of diseases. **(A)** Heatmap of gene expression levels between the DD and control groups. **(B)** Volcano plot of gene expression levels between the DD and control groups. **(C)** Heatmap of gene expression levels between the FS and control groups. **(D)** Volcano plot of gene expression level differences between the DD and control groups.

Two microarray datasets were first analyzed separately. The volcano plot and heat map in **Figures 1A–D** show the genetic differences between the two diseases. Compared with the control group, there were 2,762 differentially expressed genes in the FS group, including 1,245 downregulated and 1,517 upregulated genes. There were 1,555 differentially expressed genes in the DD group compared with the control group, including 764 downregulated and 791 upregulated genes.

### Common DEGs in DD and FS

**Figure 2:**
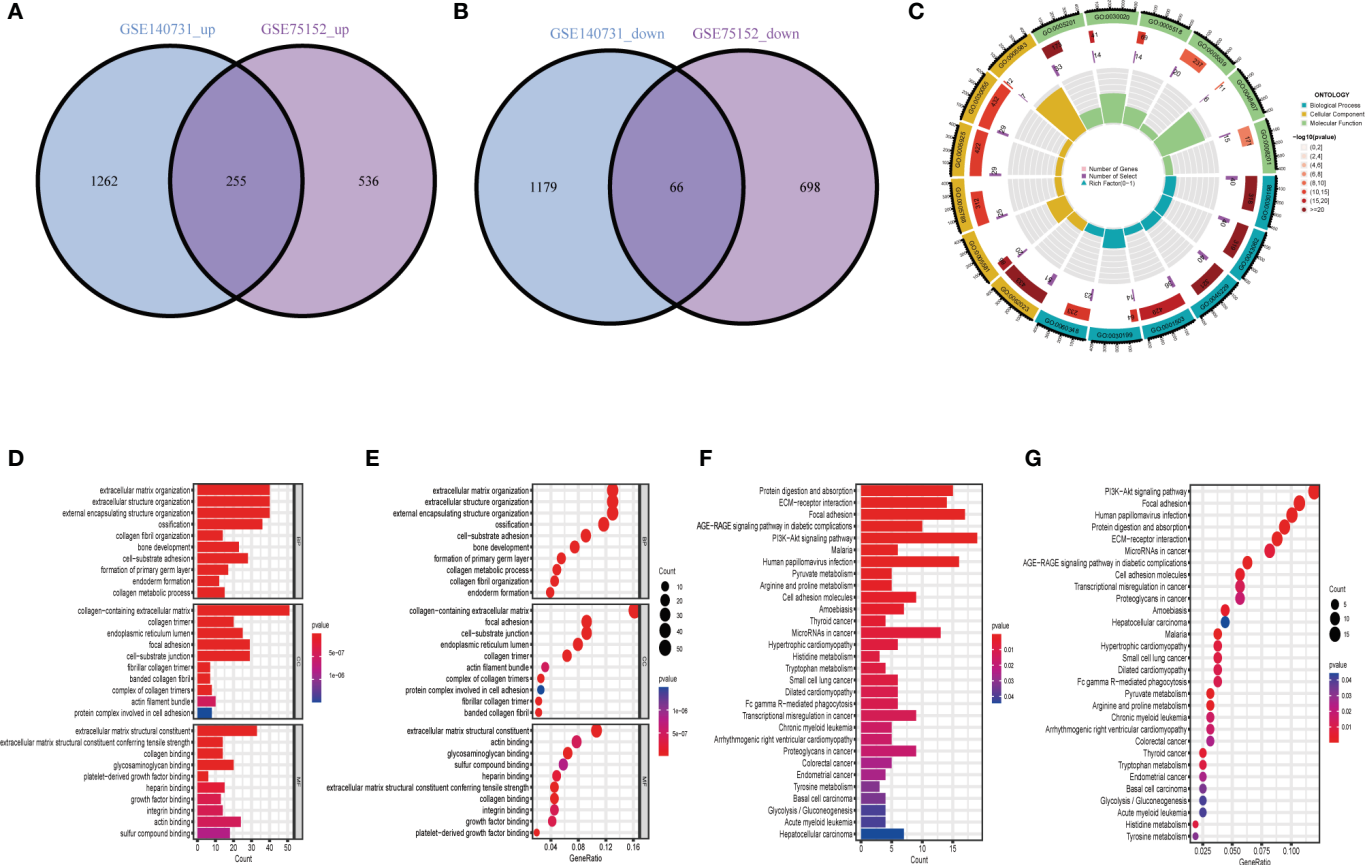
Identification and biological characteristics of common DEGs in DD and FS. **(A)** Venn diagram showing upregulated shared genes in DD and FS. **(B)** Venn diagram showing downregulated genes in DD and FS. **(C)** Circle diagram of gene enrichment numbers for each GO item among the shared genes. **(D)** GO analysis of the shared genes. **(E)** The bubble chart shows the GO enrichment and count of enrichment items among the shared genes. **(F)** KEGG analysis of the shared genes. **(G)** The bubble chart shows the KEGG enrichment and count of the shared enriched items.

There were 321 common differential genes among GSE75152 and GSE140731 DEGs, namely, 255 upregulated genes and 66 downregulated genes (**Figures 2A, B**). We analyzed GO information and KEGG pathways for these common DEGs to explore their underlying biological information. The results of GO analyses showed that in biological process (BP), genes involved in extracellular matrix structure, extracellular structure organization, encapsulating structure organization, ossification, and collagen fibril organization, among others, were especially enriched. In cellular component (CC), collagen-containing extracellular matrix, collagen trimer, and focal adhesion, among others, were represented. Molecular function (MF) items that were enriched included extracellular matrix structural constituents such as collagen binding and actin binding (**Figures 2C–E**). KEGG enrichment results showed the DEGs to be mainly enriched in protein digestion and absorption, ECM–receptor interaction, focal adhesion, and PI3K-Akt signaling pathways (**Figures 2F, G**).

### PPI network construction of common DEGs

**Figure 3:**
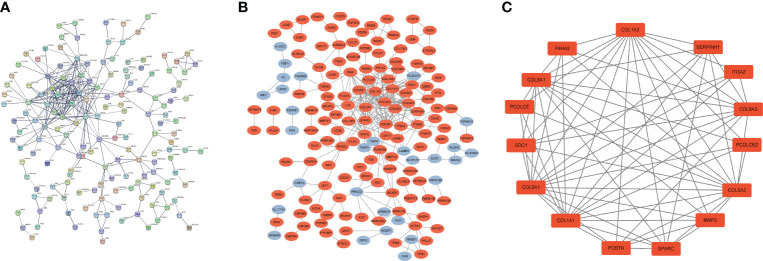
PPI network of common DEGs in DD and FS. **(A)** PPI network of shared genes (interaction score of 0.7 and PPI enrichment *p-*value < 1.0e-16). **(B)** PPI network of upregulated and downregulated shared genes (orange represents upregulated genes, and blue indicates downregulated genes). **(C)** Key clustering module of the PPI (the cluster score is 9.571).

We used common DEGs to construct a PPI network in STRING to understand potential connections between proteins, with a minimum required interaction score of 0.7 and a PPI enrichment *p*-value < 1.0e-16. This score means that the connection has a high degree of confidence (**Figure 3A**). To understand expression of interacting proteins in FS with DD, we scored protein−protein interactions and combined the PPI network with a score greater than 0.7 with whether the common DEGs were upregulated or downregulated (**Figure 3B**). We used the “MCODE” algorithm of Cytoscape to analyze and detect key clustering modules. The module consists of 15 nodes and 67 edges, and the cluster score (density multiplied by the number of members) was 9.571 (**Figure 3C**).

### Identification of hub genes and PPI network construction

**Figure 4:**
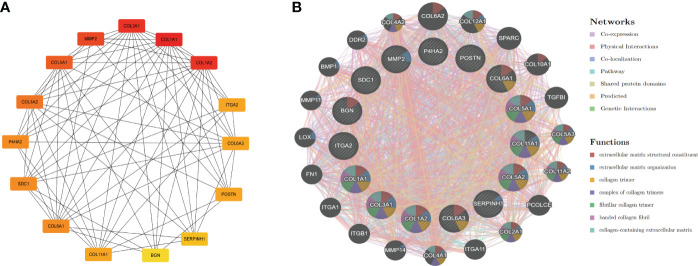
PPI network of hub genes. **(A)** PPI network of 15 hub genes (the darker the color is, the more important the gene is). **(B)** GeneMANIA predicted relationships between coexpression, shared protein domain, colocation, and pathway.

To understand interaction between hub genes, we used the “cytoHubba” algorithm to identify the top 15 hub genes. Then, the interaction of these genes was visualized, whereby a darker color indicates a more important gene (**Figure 4A**). GeneMANIA was used with the PPI to evaluate 15 hub genes and 20 interacting genes to predict relationships between coexpression, shared protein domain, colocation, and pathway aspects (**Figure 4B**). The outer circle is the predictive genes, and the inner circle is the hub genes. Network analysis showed that these genes are associated with extracellular matrix structural constituents, extracellular matrix organization, collagen trimer, collagen trimer complex, and fibrillar collagen trimer.

### GO and KEGG pathway analyses of hub genes

**Figure 5:**
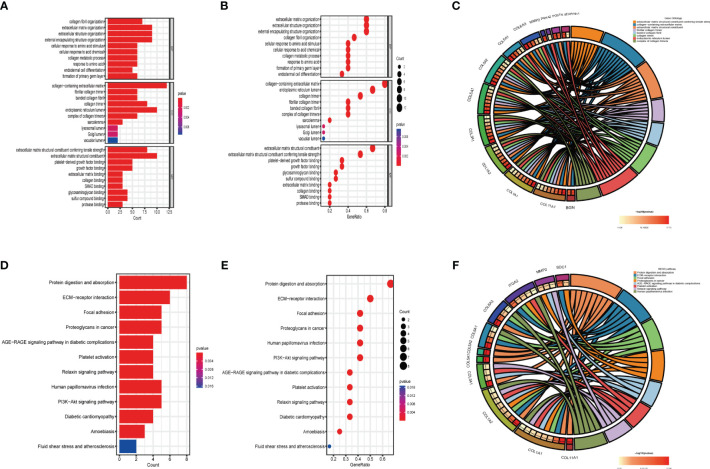
GO and KEGG analyses of hub genes. **(A)** GO analysis of hub genes. **(B)** The bubble chart shows the GO enrichment and count of enrichment items in hub genes. **(C)** The circle diagram shows the relationship between key genes and GO enrichment items. **(D)** KEGG analysis of hub genes. **(E)** The bubble chart shows the KEGG enrichment and count of enrichment items in hub genes. **(F)** The circle diagram shows the relationship between hub genes and KEGG enrichment items.

To further understand the function of hub genes in FS with DD, we enriched hub genes. According to GO analysis, collagen fibril organization, extracellular matrix organization, and extracellular structure organization, among others, were enriched in BP. Collagen-containing extracellular matrix, fibrillar collagen trimer, and banded collagen fibril, among others, were enriched in CC. Extracellular matrix structural constituents conferring tensile strength, extracellular matrix structural constituents, and platelet-derived growth factor binding, among others, were enriched in MF (**Figures 5A–C**). KEGG analysis showed that the hub genes were enriched in protein digestion and absorption, ECM–receptor interaction, and focal adhesion, among others (**Figures 5D–F**).

### Association between hub genes and TFs

**Figure 6:**
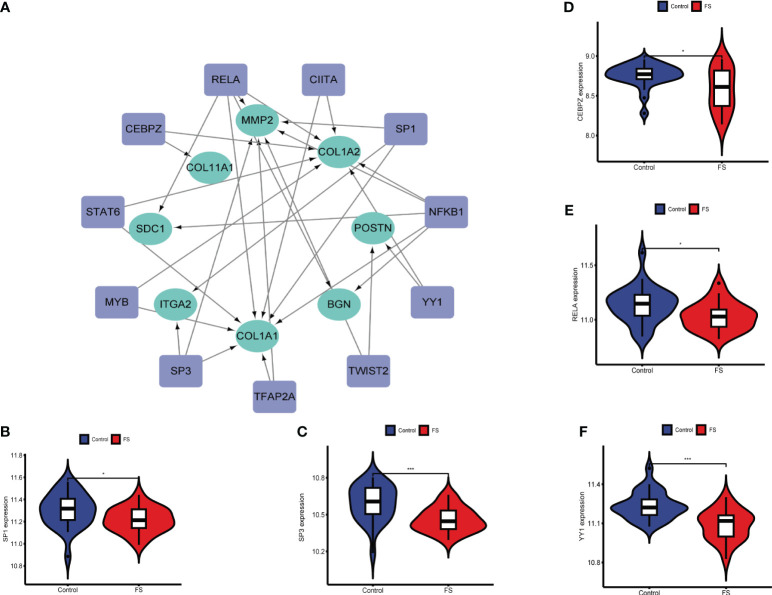
Association between hub genes and TFs. **(A)** Prediction of TF genes and their interaction network with hub genes. **(B)** Differences in expression of SP1 in the FS and control groups. **(C)** Differences in expression of SP3 in the FS and control groups. **(D)** Differences in expression of CEBPZ in the FS and control groups. **(E)** Differences in expression of RELA in the FS and control groups. **(F)** Differential expression of YY1 in the FS and control groups (*p < 0.05, ***p < 0.001).

TFs are involved in gene regulation. To explore the role of TFs, we used TRRUST to predict key TFs that affect these two diseases through hub genes. Analysis of interactions between TFs and common DEGs revealed that 11 TFs coordinated 8 common DEGs, indicating a high level of cooperation (**Figure 6A**). The top TFs were ranked according to their *p-*values and included RELA, NFKB1, CEBPZ, SP3, TWIST2, CIITA, STAT6, MYB, TFAP2A, YY1, and SP1 (**Table S1**). These findings reveal a significant relationship between common DEGs and TFs.

By analyzing expression of these TFs in GSE140731, we detected significant differences in SP3, YY1, SP1, RELA, and CEBPZ between FS patients and healthy patients (**Figures 6B–F**). This indicates that TFs influence the development of these diseases and their occurrence.

### Identification of candidate genes via machine learning

**Figure 7:**
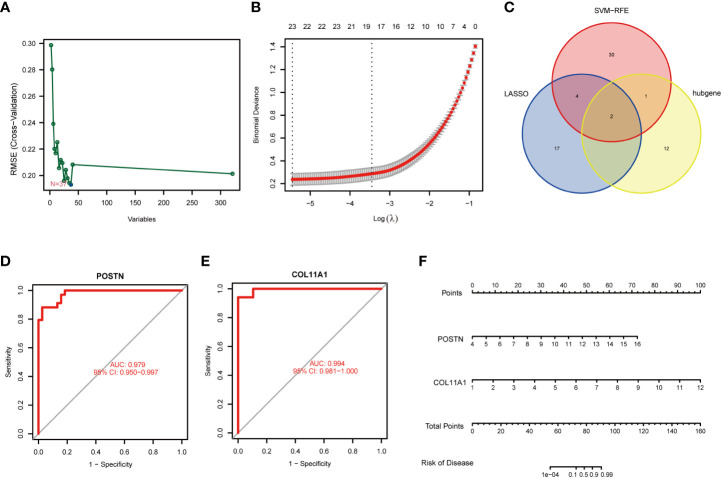
Identification of candidate genes. **(A)** Screening of key genes by LASSO regression. **(B)** Screening of key genes by SVM learning. **(C)** Candidate genes identified in the LASSO model, SVM learning, and cytoHubba algorithm. **(D)** ROC curve of the POSTN gene. **(E)** ROC curve of the COL11A1 gene. **(F)** Nomogram for diagnosis FS with DD.

LASSO regression and SVM algorithms were utilized to identify potential candidate genes of FS combined with DD. LASSO regression analysis and SVM algorithms identified 23 and 37 genes closely related to FS with DD, respectively (**Figures 7A, B**). The intersection of the 37 genes from SVM and 23 genes from LASSO identified six genes (COL11A1, POSTN, LBH, CH25H, SMOC2, and PALLD). COL11A1 and POSTN were also the top 15 hub genes (**Figure 7C**). The AUC and 95% CI of these genes were calculated by constructing an ROC curve to evaluate the diagnostic effect, as shown in **Figures 7D, E**. The results were as follows: POSTN (AUC: 0.979, CI: 0.950–0.997) and COL11A1 (AUC: 0.994, CI: 0.981–1.000). We constructed a nomogram with 2 candidate diagnostic genes (**Figure 7F**).

### Immune infiltration analysis

**Figure 8:**
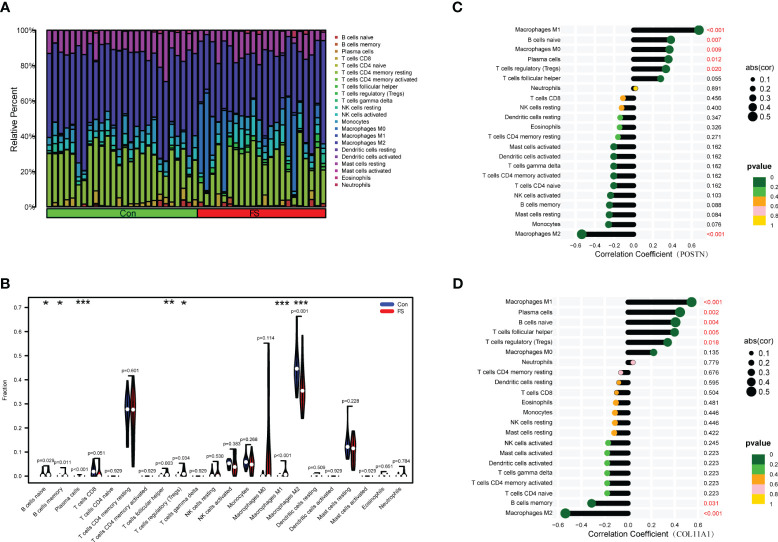
Immunocyte infiltration analysis in FS. **(A)** The bar plot shows the proportion of 22 immunocytes in GSE140731. **(B)** Levels of infiltrating immunocytes in the FS and control groups (*p < 0.05, **p < 0.01, ***p < 0.001). **(C)** The lollipop plot shows the relationship between POSTN genes and immunocytes. **(D)** The lollipop plot shows the relationship between COL11A1 genes and immunocytes.

Immunocytes may be involved in the pathogenesis of DD. However, as the relationship between immunocytes and the pathogenesis of FS is rarely reported, we conducted immune infiltration analysis to explore the effect of immunity on FS. The proportion of immunocytes in the FS group and control group is shown in **Figure 8A**. Compared with the control group, there were significant differences in levels of naive B cells, memory B cells, plasma cells, follicular helper T cells, regulatory T cells (Tregs), M1 macrophages, and M2 macrophages in the FS group (**Figure 8B**). Then, we established the relationship between candidate genes and immunocytes and observed the effects of different candidate genes on immunocytes (**Figures 8C, D**, **S1**). The results showed that expression of the POSTN gene was closely related to M1 macrophages, M2 macrophages, naive B cells, M0 macrophages, plasma cells, and regulatory T cells (Tregs) (**Figure 8C**). Expression of the COL11A1 gene was closely related to M1 macrophages, M2 macrophages, plasma cells, naive B cells, follicular helper T cells, regulatory T cells (Tregs), and memory B cells (**Figure 8D**).

## Discussion

Before 1872, FS was thought to be an inflammation of the shoulder and was labeled periarthritis by Duplay. Then, in 1934, Codman first proposed the term frozen shoulder (24). The change in terms seems to indicate that the pathological changes associated with FS have gradually shifted from inflammation to fibrosis. As the most common hereditary disease of connective tissue, DD is mainly characterized by pathological fibrosis of connective tissue (25). In 1936, Schaers (26) first reported the close relationship between FS and DD. Subsequently, increasing clinical evidence has proven the correlation between them (8, 27, 28). Furthermore, an increasing number of basic studies are also attempting to reveal the pathogenesis and potential relationship between FS and DD. Lundberg (29) reported histological similarities linking the two pathologies as early as 1969. Bunker (9) also found that there are histological similarities between FS tissue and DD tissue, confirming that both types of tissue constitute a combination of synovial inflammation and capsular fibrosis. These studies show that DD and FS share a common mechanism, but the molecular mechanism of their interaction and pathogenesis is still unclear.

Few studies have explored the susceptibility factors of DD in FS at the genetic level. Exploring the interaction of two related diseases at the genetic level may provide some inspiration for research on the two diseases. In this study, bioinformatics analysis through a public database was used to explore the mechanism of FS and DD at the gene level. First, we analyzed genetic differences in the high-throughput data of patients with FS and DD and found significant differences between patients with the two diseases and the healthy group. Subsequently, shared genes with common upregulation and downregulation were found in the two diseases, and GO and KEGG enrichment analyses were performed. These analyses showed a certain association between the two diseases at the genetic level. Third, we used the shared genes to build a PPI model and selected key clustering modules from it. The results showed an interaction at the protein level. Next, we selected the top 15 genes according to the algorithm and constructed an interactive network; similar to the key PPI model, interaction of these genes plays an important role in the pathogenesis of the two diseases. Some of the 15 hub genes have been reported to be closely related to the two diseases, such as COL1A1 and MMP2 (30–32). However, many genes have not been reported in the pathogenesis of DD or FS. Next, we examined the GO and KEGG pathways that were enriched among the hub genes, and the results showed extracellular matrix deposition, cell adhesion, and fibrosis to be closely related to these two diseases. This view is supported by previous studies (33, 34). We found some pathways that may be related to the two diseases, such as the PI3K-AKT pathway, Wnt pathway, and TGF-β pathway (**Tables S2**, **S3**). Rui Yang showed that IL-6 is upregulated in FS synovium and can promote the formation of FS fibrosis through the PI3K-AKT pathway (35). Chen reported that TGF-β1 can induce shoulder fibrosis in a rat model and that a PPAR-γ agonist can be used as a treatment (36). Similarly, some studies have suggested that the TGF-β pathway and Wnt pathway are key pathways in DD (37, 38). These studies prove the reliability of our enrichment. However, compared with other diseases, studies on the mechanism of these two diseases are scarce. Interestingly, we identified the AGE-RAGE signaling pathway, which is closely related to diabetes, in our enrichment results. Many studies have found that diabetes is a high-risk factor for FS and DD (39–42). However, the mechanism is unclear, though the AGE-RAGE signaling pathway may be a key pathway linking these diseases. Moreover, according to hub genes, we predicted TFs related to the diseases and found that some differ between the diseases. TFs play an important role in the regulation of various diseases. Our results prove that these TFs may provide an important basis for the study of FS with DD.

We identified the candidate genes POSTN and COL11A1 by machine learning. They are involved in skeletal system development, ossification, or osteoblast differentiation (43). They may play a role in FS with DD disease progression. Linda’s studies have proven that POSTN is upregulated in DD and that it can promote the transformation of fibroblasts into myofibroblasts, thus promoting progression of the disease (44). This study proves the accuracy of our bioinformatics analysis. However, studies on the effect of POSTN on FS have not been reported.

Since DD can induce FS, we wanted to determine the effect of candidate genes on FS immune cells. Then, we found that immunocytes are involved in the pathogenesis of FS. Although there are few studies on the mechanism of the effect of immunocytes on FS, the role of inflammation in the two diseases has been proven; thus, immunocytes are very important for progression of the disease. Exploring the mechanism of immune cells in FS may be a breakthrough in the study of disease mechanisms. The important role of imbalance between M1 and M2 macrophages in fibrotic diseases has been demonstrated by many studies (45, 46). Our study also found that imbalance between M1 and M2 macrophages is involved in the occurrence and development of FS. Finally, to further explore the important role of immune cell infiltration and candidate genes in FS, we assessed the correlation between them. Our results show that the candidate genes can affect disease progression by regulating immunocytes.

## Limitations of the study

**Figure 9:**
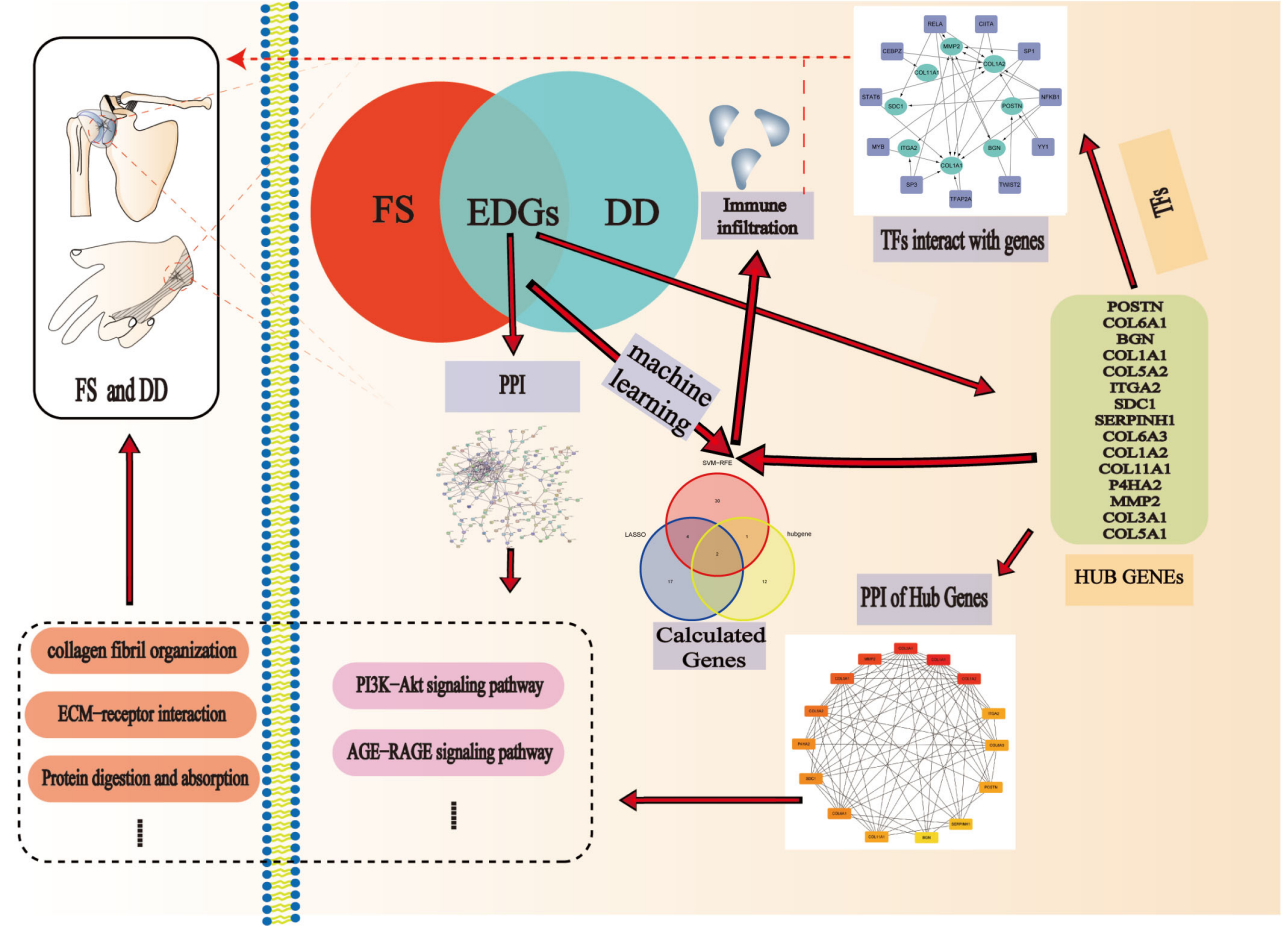
Pathogenesis of the two diseases at the genetic level.

Through the interactive study of FS and DD, we explored the pathogenesis of the two diseases at the genetic level (**Figure 9**). The results suggest new implications for the study of the pathogenesis of the two diseases. However, there are still some shortcomings in our study. First, the key genes and pathways found were not verified by experiments. However, some genes and related pathways have been confirmed in previous studies, which proves the reliability of this study. The focus of our subsequent studies will be to demonstrate the effects of these pathways and genes on FS. Due to the lack of high-throughput FS and DD datasets, the results cannot be verified by other datasets. More high-throughput sequencing data are needed to verify our results. Finally, the larger the sample size is, the more accurate the results of bioinformatics analysis is. It is hoped that there will be larger sample size datasets in the future.

## Conclusion

We identified the PPI network, 15 hub genes, and two immune-related candidate genes (POSTN and COL11A1) using bioinformatics analysis and machine learning algorithms. These genes have the potential to serve as diagnostic candidate genes for FS in DD patients. Furthermore, our study reveals disorder of immunocytes in FS. We clarified the potential mechanism of the relationship between FS and DD at the genetic level, providing a new idea for future research on DD and FS.

## Data availability statement

Publicly available datasets were analyzed in this study. This data can be found here: https://www.ncbi.nlm.nih.gov/geo/ under the accession numbers GSE75152 and GSE140731. The GSE75152 and GSE140731 datasets were obtained from the GEO database.

## Author contributions

GS and YO designed this study. SC and TW conducted the literature searches and management. YO, YT, and HF were responsible for the data management and statistical analysis. SC and YO interpreted the findings. YO wrote the manuscript. The manuscript was revised by all authors, and the final version was approved by all.

## Supplementary material

Supplementary Figure 1
**(A–M)** Correlation between candidate genes and immunocytes.

Supplementary Table 1 Key TFs predicted according to hub genes.

Supplementary Table 2 KEGG analyses of hub genes.

Supplementary Table 3 GO analyses of hub genes.
