# Supplementary figures and images for: Exploring the potential relationship between frozen shoulder and Dupuytren’s disease through bioinformatics analysis and machine learning
Source: Front Immunol. 2023 Aug 31;14:1230027. doi: 10.3389/fimmu.2023.1230027 (PMC10500125; doi:10.3389/fimmu.2023.1230027)

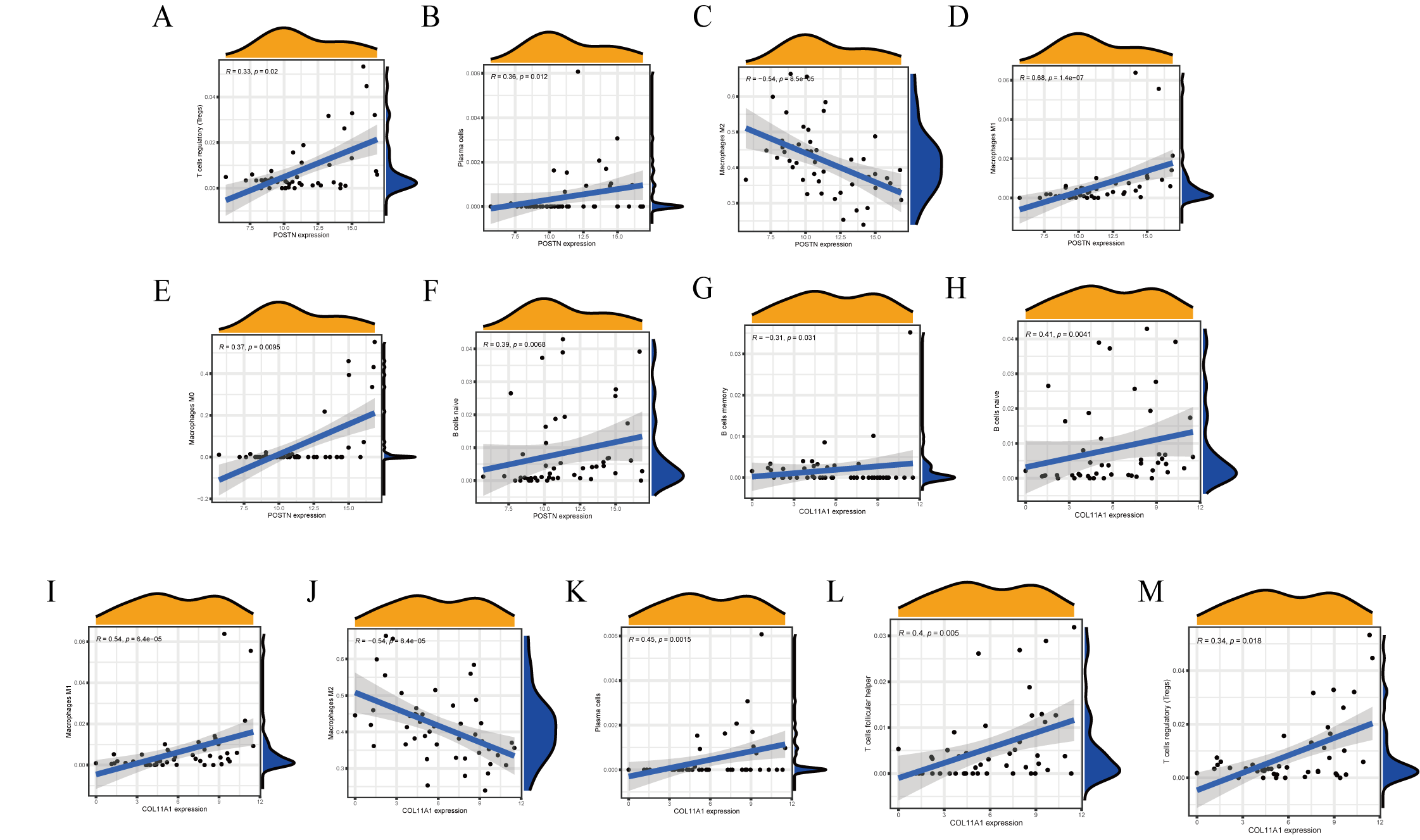

Supplement: Supplementary Figure 1 — (A–M) Correlation between candidate genes and immunocytes. [file Image_1.tif]
